# Molecular Weight-Dependent, Flexible Phase Behaviors of Amphiphilic Block Copolymer/Additive Complexes in Aqueous Solution

**DOI:** 10.3390/polym13020178

**Published:** 2021-01-06

**Authors:** Jong Dae Jang, Young-Jin Yoon, Sang-Woo Jeon, Young Soo Han, Tae-Hwan Kim

**Affiliations:** 1 Quantum Beam Material Science Research Division, Korea Atomic Energy Research Institute, 1045 Daedeok-daero, Yuseong-gu, Daejeon 34057, Korea; jdjang@kaeri.re.kr (J.D.J.); yshan@kaeri.re.kr (Y.S.H.); 2 Department of Applied Plasma & Quantum Beam Engineering, Jeonbuk National University, 567 Baekje-daero, Deokjin-gu, Jeonju 54896, Korea; dudwls5751@jbnu.ac.kr (Y.-J.Y.); ejn00013@jbnu.ac.kr (S.-W.J.); 3 Department of Quantum System Engineering, Jeonbuk National University, 567 Baekje-daero, Deokjin-gu, Jeonju 54896, Korea

**Keywords:** Pluronic block copolymer, self-assembly, phase behavior, highly ordered nanostructure, closed loop-like phase behavior

## Abstract

Pluronic amphiphilic block copolymers, well known to have a phase behavior can be controlled by external conditions, have a wide range of potential for applications such as nanotemplates or nanobuilding blocks. However, the phase behaviors of Pluronic block copolymer/additive complexes with highly ordered phases have not been fully investigated. Here, we report the unusual molecular weight-dependent self-assembly of Pluronic block copolymer/additive complexes. Depending on the temperature and additive, Pluronic P65 block copolymer with a lower molecular weight showed the closed loop-like (CLL) phase behavior with the disorder-order-disorder-order phase transition in aqueous solution, whereas Pluronic P105 and P85 block copolymers with higher molecular weights underwent highly ordered continuous phase transitions with face centered cubic (FCC), hexagonal, and lamellar phases. It is expected that the specific phase behavior of the block copolymer/additive complex can be applied in optical devices such as nanotemplates or optical sensors for a highly ordered superlattice. Furthermore, this study provides a new route to control the phase behavior of the block copolymers without a complicated process.

## 1. Introduction

The formation of highly ordered lattices within confined nanostructures is an attractive phenomenon with potential applications in nanoscale systems [1,2,3]. In general, amphiphilic molecules (such as a surfactants and amphiphilic block copolymers) can self-assemble into various nanostructures [4,5,6,7,8,9,10,11], providing multiple possibilities for application as nanobuilding blocks, nanotemplates, and nanocarriers in aqueous solutions. Pluronic block copolymers range from 1000 to 15,000 g/mol in molecular weight and are well-known for their amphiphilic properties. A Pluronic triblock copolymer consists of a hydrophobic poly(propylene oxide) (PPO) center block surrounded by two hydrophilic poly(ethylene oxide) (PEO) side blocks (PEO_m_-PPO_n_-PEO_m_). Although the phase behaviors of Pluronic block copolymers can be controlled by the external conditions [12,13,14,15,16], the general understanding of the phase behaviors of amphiphilic molecules with different molecular weights is still insufficient due to the diversity of amphiphilic molecules and external conditions. To understand how the molecular weight of Pluronic block copolymers affects their structure, we investigated the phase behaviors of Pluronic block copolymers of different molecular weights, with a hydrophilic mass fraction (*f*) of 0.5, where the structure was critically affected by the hydrophobicity [17,18] (The Pluronic block copolymers P105 (PEO_37_-PPO_58_-PEO_37_), P85 (PEO_26_-PPO_40_-PEO_26_), and P65 (PEO_20_-PPO_30_-PEO_20_) with molecular weights of 6500 g/mol, 4600 g/mol, and 3500 g/mol, respectively, were studied). This is a control method for determining the phase behavior of polymers and is one of our leading experimental methods. Pluronic block copolymers with *f* = 0.5 were used because their steric conformations were sensitive to changes in external conditions, such as temperature and additives [19,20], and it is easy to control the phase behavior of Pluronic block copolymers by varying the temperature and additives. Here, we added a small derivative molecule bearing an aromatic functional group and a hydroxide group, viz. 5-methylsalicylic acid (5mS). Because the 5mS molecule has an asymmetric amphiphilicity (with a relatively large hydrophobic portion (aromatic group) and small hydrophilic portion (hydroxide group)) and temperature dependent solubility in water (the solubility of 5mS increases with temperature) as well as a strong reactivity for binding with amphiphilic molecules, the steric conformation of Pluronic block copolymer can be easily modified depending on temperature and the concentration of 5mS molecules, thus allowing the formation of various phase behaviors. The phase behaviors of the Pluronic block copolymer/5mS complexes were investigated by small-angle X-ray scattering (SAXS) analysis, because the SAXS technique could be applied to investigate the phase behavior of a variety of soft matters, including block copolymers, surfactants, and biomaterials [21,22,23].

The SAXS analysis and visual inspection of the Pluronic block copolymer/5mS complexes revealed that, depending on temperature, both the ordered and disordered structures were formed, corresponding to solid-like gel and fluidic phases, respectively. While both Pluonic P105 and P85 formed solid-like gels continuously upon changing the temperature and concentration of additives, Pluonic P65 changed repeatedly between the fluidic and solid-like gel phases. SAXS analyses of the Pluronic block copolymer/5mS complexes revealed an order-order phase transition (face-centered cubic (FCC)-hexagonal-lamellar) for the Pluronic P105- and P85-5mS complexes, and disorder-order-disorder-order phase transition (closed loop-like, CLL) for the Pluronic P65-5mS complex. It should be noted that, depending on the temperature and concentration of the 5mS additive, the Pluronic P65 block copolymer with a lower molecular weight showed a CLL phase behavior with the disorder-order (hexagonal)-disorder-order (lamellar) phase transition in aqueous solution, whereas Pluronic P105 and P85 block copolymers with higher molecular weights underwent highly ordered continuous phase transitions with FCC, hexagonal, and lamellar phases. This study provides fundamental information on the phase behavior of amphiphilic molecules and their dependence on molecular weight in the phase transition, including the unique CLL phase behavior.

## 2. Methods/Experimental

### 2.1. Materials

Pluronic amphiphilic block copolymers P105 (PEO_37_-PPO_58_-PEO_37_, molecular weight = 6500 g/mol), P85 (PEO_26_-PPO_40_-PEO_26_, molecular weight = 4600 g/mol), and P65 (PEO_20_-PPO_30_-PEO_20_, molecular weight = 3500 g/mol) were purchased from BASF (Ludwigshafen, Germany). 5-Methylsalicylic acid (5mS) was purchased from Tokyo Chemical Industry (Tokyo, Japan). All chemicals were used as received. H_2_O was purified by a Millipore Direct Q system (Bay city, MI, USA) immediately before use.

### 2.2. Sample Preparation

All samples were prepared by simply mixing the Pluronic amphiphilic block copolymer and 5mS in aqueous media. The concentration of the Pluronic block copolymers P105, P85, and P65 was 35 wt %, 33 wt %, and 40 wt %, respectively, whereas the concentration of 5mS was varied in the range of 0–7.5 wt % at 2.5 wt % intervals for the P105 solution and 2 wt % intervals for the P85 and P65 solutions. The Pluronic block copolymer-5mS complexes were agitated at 60 °C followed by vortex mixing to ensure homogeneity.

### 2.3. Polarized Optical Microscopy (POM)

POM measurements were performed to check the phase transitions of the Pluronic block copolymer/5mS complexes using the POM instrument (Model: Olympus BX-53; ToKyo, Japan).

### 2.4. Small-Angle X-ray Scattering Measurements

SAXS measurements were performed on the 4C and 9A beamlines at the Pohang accelator laboratory. X-ray beams with a wavelength of 0.735 Å and an energy resolution (ΔE/E) of approximately 2 × 10^−4^ were obtained from a double crystal monochromator with a Si(111) crystal at the Pohang Accelerator Laboratory (Pohang, Korea). Scattered X-ray intensities were measured by a 2-dimensional CCD camera (SX165, Rayonix; Evanston, IL USA). Sample cells with a thickness of 0.8 mm were used, and the windows on both sides were sealed with Kapton films. The sample-to-detector distance was 2 m, covering the *q* range of 0.012–0.17 Å^−1^. The *q* range was calibrated using silver behenate [24].

## 3. Results and Discussion

The molecular weight of an amphiphilic Pluronic block copolymer can affect its phase transitions, along with previously known external factors, such as concentration, additive, and temperature. Additionally, the phases of the Pluronic block copolymers with *f* = 0.5 are easily transformed without any special perturbation. The Pluronic block copolymers with different molecular weights of 3500 g/mol, 4600 g/mol, and 6500 g/mol were termed Pluronic P65, P85, and P105, respectively. Since *f* = 0.5 for all the studied Pluronic block copolymers, in this study, the phase behavior of block copolymers should be dependent on their molecular weights, which are determined by their chain lengths. Since Pluronic block polymers with a variety of phases have potential applications as nanotemplates and optoelectronics in the nanoscience field [25,26], it is important to understand their phase behaviors in terms of the molecular weights of the amphiphilic molecules, temperature, and additives. In this study, the used additive, mS, has a hydrophobic phenyl moiety that makes up a significant portion of the 5mS molecule, and an increase in the 5mS content in the complex further reduces *f* because the total hydrophobicity increases. In addition, since the increase in temperature of the complex leads increased solubility of 5mS, the experiments were systemically designed to study the effects caused by changes in temperature and 5mS concentration [27]. The concentrations of the Pluronic block copolymer were 35 wt %, 33 wt %, and 40 wt % for Pluronic P105, P85, and P65 with 5mS additive concentrations of 2.5 wt %, 2 wt %, and 2 wt %, respectively. The Pluronic block copolymers showed that the Pluronic P105 and P85 solutions formed a solid-like gel phase, while Pluronic P65 solution formed a fluidic phase (the low molecular weight of Pluronic P65 prevented the formation of a solid-like phase under ambient conditions). Although 5mS is poorly soluble in water at room temperature, it dissolves upon the addition of the Pluronic block copolymers. To investigate the nanostructures, the complexes were prepared by simply mixing both the Pluronic block copolymer and 5mS.

**Figure 1:**
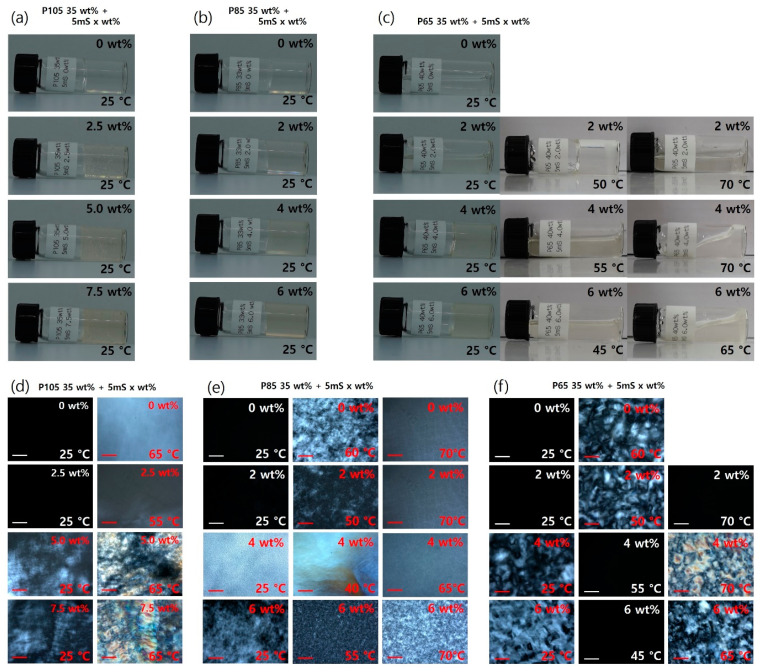
Visual inspection of the Pluronic block copolymer-5mS complexes at different temperatures: (**a**) P105-5mS complexes at 25 °C, (**b**) P85-5mS complexes at 25 °C, and (**c**) P65-5mS complexes at 25 °C and 45–70 °C). Polarized optical microscopy (POM) images at different temperatures under the crossed polarizer condition: (**d**) P105-5mS complexes at 25, 55, and 65 °C, (**e**) P85-5mS complex at 25 °C and 40–70 °C, and (**f**) P65-5mS complex at 25 °C and 45–70 °C). The scale bar in (**d**–**f**) is 100 μm.

The phase transitions of the Pluronic-5mS complexes were investigated by simple visual inspection of the samples and polarized optical microscopy (POM) (Figure 1). Generally, when the block copolymers in aqueous solution self-assemble into an ordered structure, they form a solid-like gel phase with high viscosity. Therefore, we confirmed the phases of the Pluronic block copolymer-5mS complexes at different temperatures and 5mS concentrations by visual inspection. While all the P105 and P85-5mS complexes formed solid-like gel phases over the entire temperature range studied (Figure 1a,b), confirming the formation of the ordered phase, the viscosities of the P65-5mS complexes changed with temperature and 5mS concentration, indicating disorder-order or order-disorder phase transition (Figure 1c). The POM images obtained under the crossed polarizer condition simply provides information on the directionality of the sample that forms a phase, such as a hexagonal or lamellar phase. Polarized light cannot pass through a polarizing filter with directionality perpendicular to its oscillation direction (called crossed polarized condition). However, we can observe polarized light as a form of birefringent texture when the sample with an anisotropic ordered phase is placed between the polarizers because of the different refractive indices between the original and orthogonal directions in the sample. Therefore, we expected that the hexagonal or lamellar phase showed birefringent texture, while the cubic phase (such as BCC and FCC) did not show any texture. Figure 1d–f show the POM images for the P105-5mS, P85-5mS complexes, and P65-5mS complexes, respectively, at different temperatures and 5mS concentrations. Except for all Pluronic block copolymer-5mS complexes with 5mS concentrations of 0 and 2% at 25 °C and P65-5mS complexes with 5mS concentrations of 2 wt % (at 70 °C), 4 wt % (at 55 °C), and 6 wt % (at 45 °C), the Pluronic block copolymer-5mS complexes showed birefringent-texture images, indicating the formation of an anisotric ordered phase, such as 2D hexagonal or lamellar structures.

To investigate the various phase behaviors with more detailed conformation, SAXS experiments were performed. SAXS analysis is useful for observing the nanostructure because the SAXS intensities reflected the intraparticular and interparticular interferences of the samples. To confirm the results of the visual inspections and POM analyses, therefore, SAXS analyses performed by increasing the temperature from 15 °C to 70 °C at intervals of 5 °C, and holding for 10 min at each temperature to form nanostructure phases of the complexes.

**Figure 2:**
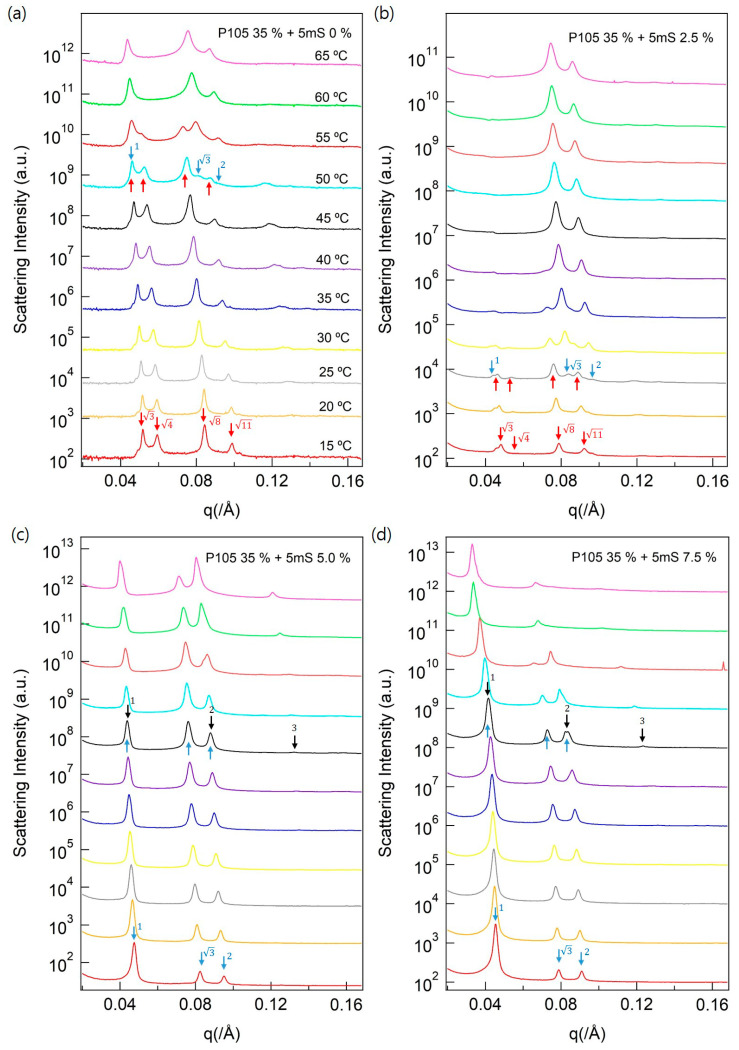
Small-angle X-ray scattering (SAXS) intensities of the P105-5mS complex in aqueous solution. The complexes of (**a**) P105 35%-5mS 0.0 %, (**b**) P105 35%-5mS 2.5 %, (**c**) P105 35%-5mS 5.0 %, and (**d**) P105 35%-5mS 7.5 % were conducted depending on a various temperature. The red, light blue, and black arrows indicate the SAXS signals of the FCC, hexagonal, and lamellar phases, respectively.

The Pluronic P105 (35 wt % concentration) solution formed the solid-like gel phase, which is confirmed by the visual inspection. The sample was not influenced by changes in the external conditions, such as temperature increase and addition of 5mS. At the same time, the SAXS intensities of the P105 solution, which revealed strong interparticular interferences (Figure 2a) with the strong interparticular interferences of the high-intensities, where the peak ratio is $\sqrt{3},\sqrt{4},\;\sqrt{8},$ and $\sqrt{11}$ (red arrow in Figure 2a). The peak ratios of $\sqrt{3},\sqrt{4},\;\sqrt{8},$ and $\sqrt{11}$ can be indexed to the FCC nanostructure by the Bragg peak index (Figure 2a) [28,29,30]. Although the sample phases of the P105-5mS complex were visually unchanged, the SAXS signal patterns slowly changed from the FCC nanostructure to the hexagonal phase (peak ratio of $1:\sqrt{3}:2$) with an increase in the temperature above 50 °C (light blue arrow in Figure 2b,c) [28,31,32]. The SAXS measurements showed that the interesting phase behavior in the nanoscale region differed from a visual inspection, which revealed only a solid-like gel. However, Figure 2b shows that the first peak of the SAXS signal of the hexagonal phase shifts to the left side, and slowly disappeared. This phenomenon is due to the influence of the interparticular interference on the intraparticular interference region. The first peak of the interparticular interference is hidden by that of the intraparticular interference. When the concentration of 5mS was 5 wt % and 7.5 wt % and the temperature was above 55 °C, the SAXS intensities showed the lamellar structure with a peak ratio of 1:2:3 (black arrow in Figure 2c,d) [28,33,34]. Herein, all the P105-5mS complexes showed single-multi-single-phase behavior in the temperature range 15–65 °C. Since the FCC phase to the hexagonal phase, the SAXS signals with the FCC phase slowly decreased, and the signals with the hexagonal phase slowly increased with increasing temperature (Figure 2a,b). The hexagonal to lamellar phase behavior also followed the same case (Figure 2c,d). Therefore, the phase behaviors of the P105-5mS complexes show that 5mS concentration effectively controls the phase behavior of the Pluronic P105 block copolymer.

**Figure 3:**
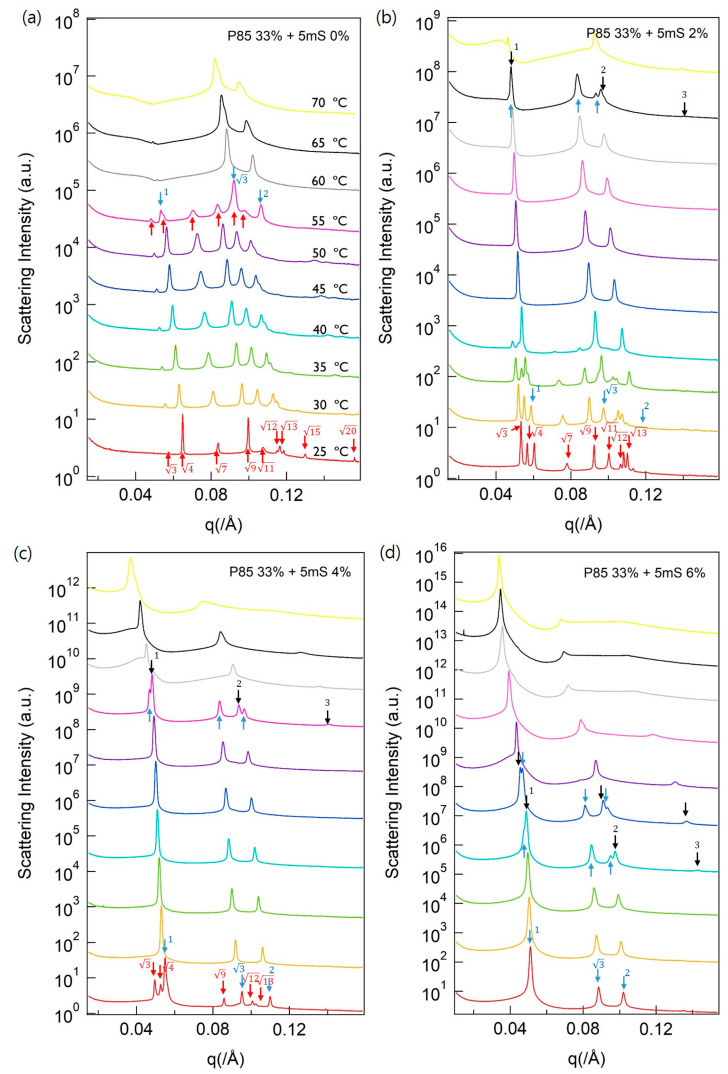
SAXS intensities of the P85-5mS complex in aqueous solution. (**a**) P85 35%-5mS 0.0 %, (**b**) P85 35%-5mS 2.0 %, (**c**) P85 35%-5mS 4.0 %, and (**d**) P85 35%-5mS 6.0 % were conducted depending on a various temperature. The red, light blue, and black arrows indicate the SAXS signals of the FCC, hexagonal, and lamellar phases, respectively.

In the case of the Pluronic P85 solution, a solid-like gel was observed by visual inspection at 33 wt %. To investigate the nanostructures of the P85-5mS complexes and their phase behaviors, SAXS measurements were performed at different temperatures and 5mS concentrations. At 25 °C, the P85 solution adopted a highly ordered FCC nanostructure. SAXS measurements revealed peak ratios of $\sqrt{3},\sqrt{4},\;\sqrt{7},$ $\sqrt{9},\;\sqrt{11},\sqrt{12},\;\sqrt{13},\;\sqrt{15}$, and $\sqrt{20}$ for the P85 solution (red arrows in Figure 3a). When the temperature was increased to 60 °C, the structure was changed from the FCC to the hexagonal phase, where the Bragg peak was indexed with the ratio of $1:\sqrt{3}:2$ (light blue arrows in Figure 3a). However, the height of the first peak is very small, where the intensity measured the P85 solution at 60 °C, 65 °C, and 70 °C. This is because it reflects the result of the intraparticular interference. The SAXS intensity (I(q)) is defined as I(q) = P(q) (intraparticular interference) × S(q) (interparticular interference). The intraparticle and interparticle interferences can be easily changed by varying the external conditions; therefore, the height of the SAXS signal can differ depending on the external conditions. In Figure 3b–d, the 5mS can be effectively controlled the phase behavior of the P85, similar with the phase behaviors of the P105-5mS complex. 5mS led to a more efficient phase transition and induced a lamellar phase with a peak ratio of 1:2:3 (Figure 3c,d). The P85 solution has a relatively low molecular weight compared to the P105 solution; therefore, the low molecular weight can easily induce a phase transition. As the temperature and 5mS concentration increased, the P85 and P105 solutions underwent a phase transition and existed as a two-phase system under the same conditions. The change in the SAXS intensities shows the process of phase transition for the multi- and two-phase P85-5mS and P105-5mS complexes. For example, the phase transition process of P105 solution in the range of 45–60 °C was indicated by variations in the intensities of the Bragg peaks, and the changing SAXS intensities revealed the change from the FCC to the hexagonal phase (multi-phase system), whereas a single phase existed at other temperatures (Figure 2a). In the case of P85 solution, the phase transition process was observed in the temperature range of 50–60 °C (Figure 3a).

**Figure 4:**
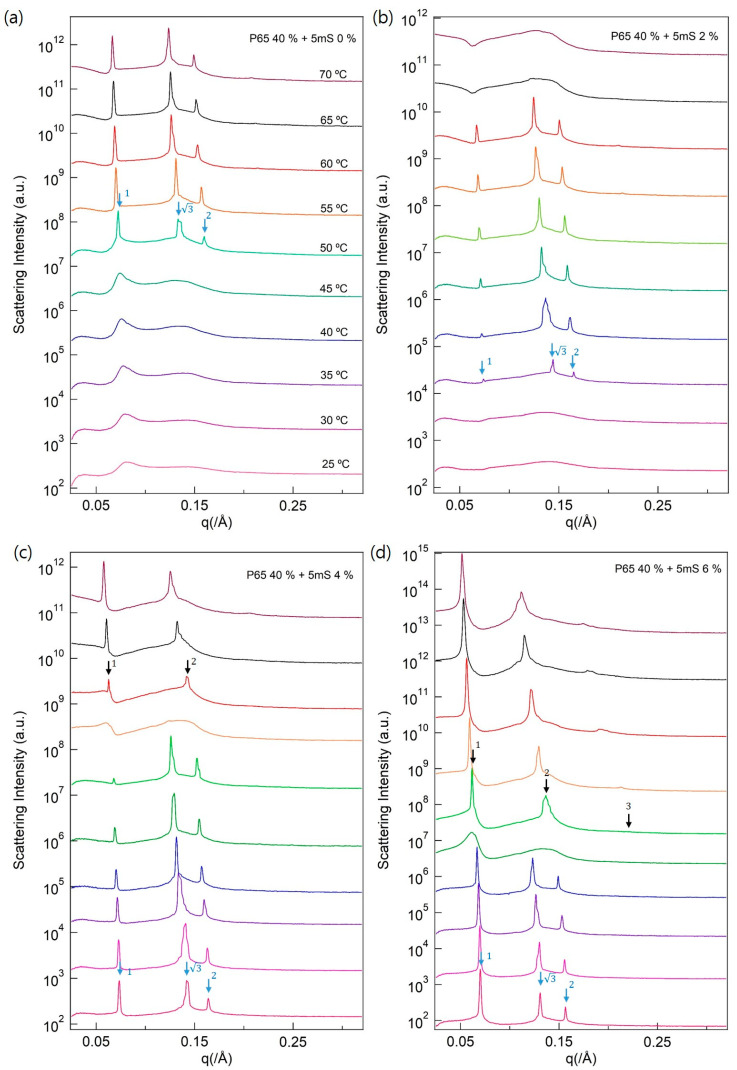
SAXS intensities of the P65-5mS complex in aqueous solution. (**a**) P65 35%-5mS 0.0 %, (**b**) P65 35%-5mS 2.0 %, (**c**) P65 35%-5mS 4.0 %, and (**d**) P65 35%-5mS 6.0 % were conducted depending on a various temperature. The red, light blue, and black arrows indicate the SAXS signals of the FCC, hexagonal, and lamellar phases, respectively.

The P65 solution formed a fluidic phase at 40 wt %. The interparticular interference revealed by the SAXS intensities indicated that the P65 solution formed supramolecular structures (Figure 4a). The visual inspection of the P65 solution showed that the viscosity increased with the increase in the temperature. Consequently, the SAXS intensities showed stronger interparticular interference than at 25 °C, and Bragg peaks appeared below 50 °C. The Bragg peaks have a peak ratio of $1:\sqrt{3}:2$, which correspond to the hexagonal phase. Herein, the P65 solution was visually changed from a fluidic phase to a solid-like gel. Interestingly, the phase transition P65 solution occurred at the same time at the nano-region (isotropic to hexagonal phase) and visual-region (fluidic to solid-like gel phase). In accordance with previous studies, regarding the phase behavior of P65, the temperature largely influenced the Pluronic block copolymer molecular conformation in aqueous solutions. At 25 °C, the intermolecular interference peak at *q* = 0.07 Å^−1^ is broad, but it becomes sharp by increasing the temperature until 50 °C, indicating that the P65 solutions were randomly distributed in the aqueous solution (isotropic phase), which is a visibly fluidic phase. In addition, the Bragg peaks observed in the temperature range 50–70 °C suggest the existence of a spontaneously ordered hexagonal phase in the aqueous solution, which visibly changed to a solid-like gel phase.

Regarding the P65-5mS complex, the temperature and 5mS concentration were varied at the same time to effectively control the phase (Figure 4a,b) and induce a phase behavior transition (Figure 4c,d) by adding the 5mS additive. The P65 sample with 2 wt % 5mS had the appearance of a fluidic phase at 25 °C (Figure 4b). The phase behavior of the P65-5mS complex is similar to that of the P65 solution, but the Bragg peaks that indicate the hexagonal phase appeared at a lower temperature for the sample containing the 5mS additive. Therefore, we conclude that the 5mS additive is useful in controlling the phase behavior of Pluronic P65. However, the SAXS signal intensities of the hexagonal phase increased at temperatures above 35 °C, reflecting a quantative increase in hexagonal phase of the sample. At 65 °C, the phase was visibly changed to a fluidic phase, and the SAXS signal showed an isotropic phase. In addition, at a 5mS concentration of 4 wt % (Figure 4c), the lamellar phase (peak ratio of 1:2:3) appeared at temperatures above 60 °C after the hexagonal and isotropic phases, revealing that the 5mS additive led to the formation of a new phase in the P65-5mS complex. The temperature-dependent SAXS intensities of the P65-5mS complex at the 5mS concentrations of 4 wt % and 6 wt % show an order-disorder-order phase transition, which corresponds to the CLL phase behavior (Figure 4c,d). Whereas the P65-5mS complex sample showed a fluidic phase by visible inspection over the temperature range 52–56 °C, the phase changed again to the viscous gel phase at higher temperature ranges (Figure 4c). This is a very interesting phase behavior of the block copolymer complex because the CLL phase behavior is rarely observed for amphiphilic molecules, and the phase transition did not occur via a multi-phase system in the P105-5mS and P85-5mS complexes. In the P65-5mS complex, there were four distinguishable phases in the temperature range 25–70 °C, and the Pluronic block copolymer P65 sensitively responded to the changes in the temperature and concentration of the additive by adopting a single isotropic, hexagonal, isotropic, and lamellar phase. In the P105-5mS and P85-5mS complexes, considering the molecular weight dependency, the phase behavior of the P65-5mS complex was expected to be more effective than those of P105 and P85 by adding 5mS additive because of the relatively low molecular weight of Pluronic P65. However, the phase behavior of the P65-5mS complex showed different patterns with CLL phase behavior. For the P65-5mS complex containing high concentration of P65, the SAXS measurements did not show sharp peaks at low temperatures. With increasing temperature, however, distinct interaction peaks appeared. Within the temperature range in which sharp Bragg peaks are observed, the visually observed formation of a solid-like gel indicates that the viscosity of the P65-5mS complex is related to the phase transitions.

**Figure 5:**
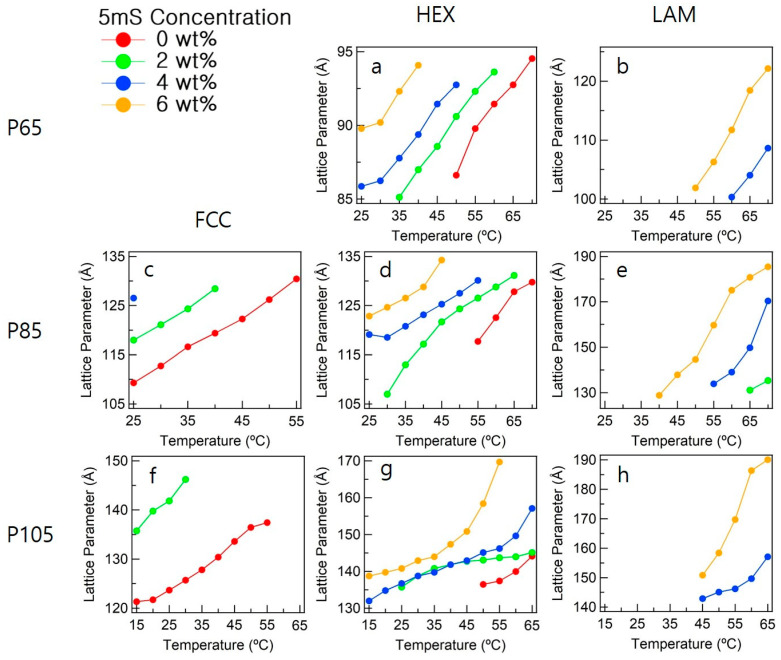
Lattice parameters of P65-5mS, P85-5mS, and P10-5mS complexes. The inter-planer distances of D_100_ distances of (**a**) Hexagonal and (**b**) lamellar of P65-5mS complex, a d_111_ distance of (**c**) FCC of P85-5mS complex, d_100_ distances of (**d**) Hexagonal and (**e**) lamellar of P85-5mS complex, a d_111_ distance of (**f**) FCC of P105-5mS complex, and d_100_ distances of (**g**) Hexagonal and (**h**) lamellar of P105-5mS complex displayed varied on a 5mS concentration, respectively.

The phase behaviors of the Pluronic block copolymers with *f* = 0.5 were investigated by SAXS measurements. The three Pluronic block copolymers with different molecular weights were used to investigate the phase behavior, which was observed to depend on the temperature and 5mS concentration. The phase behaviors of the Pluronic block copolymers depended on their *f*, which is subject to the combined effect of the temperature increase and addition of 5mS. The lattice parameter (where is the inter-planar distance d_111_ for the FCC and d_100_ for the hexagonal and lamella) increased with the increase in the temperature. This observation can be explained by the increase in the conformational entropy of the Pluronic block copolymer-5mS complex nanostructures (Figure 5). The *f* of the Pluronic block copolymer-5mS complex decreases as the concentration of the additive rises. As the hydrophobic interactions become stronger, the temperature range of the phase transition decreases, and the visual inspections were consistent with the SAXS measurements.

Figure 5 displays the lattice parameters with their structural properties. Whereas P65 adopted the hexagonal and lamellar structures, P105 and P85 existed as FCC, hexagonal (HEX), and lamellar (LAM) structures. For the majority of the structures, the structure-forming temperatures decreased with the increase in the 5mS concentration, a trend that is distinct for the hexagonal and lamellar structures. In addition, the lattice parameters increased with the increase in the 5mS concentration at a given temperature. For example, the FCC lattice parameters of P85 increased from 109 Å to 130 Å in the temperature range of 25–55 °C, whereas the ones of P105 increased from 121 Å to 147 Å in the temperature range of 15–55 °C. In the temperature range of 25–70 °C, the hexagonal lattice parameters of P65 increased from 85 Å to 95 Å, whereas those of P85 increased from 107 Å to 135 Å. The hexagonal lattice parameters of P105 increased from 131 Å to 170 Å in the temperature range of 15–65 °C. The lamellar lattice parameters of P65 increased from 85 Å to 95 Å in the temperature range of 50–70 °C, those of P85 increased from 107 Å to 135 Å in the temperature range of 40–70 °C, and the ones of P105 increased from 131 Å to 170 Å in the temperature range of 45–65 °C.

**Figure 6:**
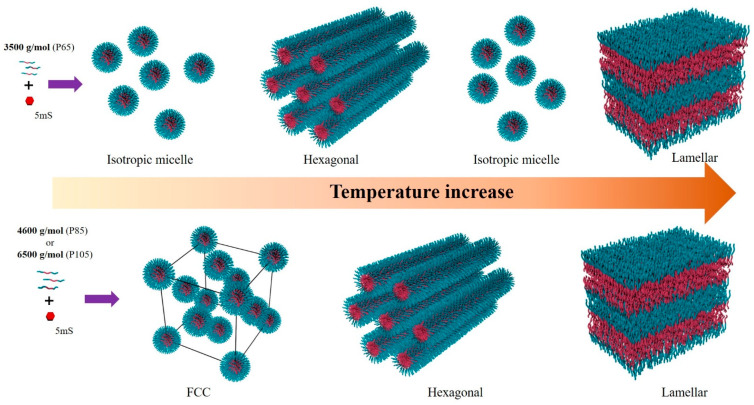
Schematic phase behaviors of Pluronic block copolymer/additive complexes depending on their molecular weight.

The three Pluronic block copolymers with different molecular weights existed in four types of structural formations with isotropic, cubic, hexagonal, and lamellar phases. In the case of P105- and P85-5mS complexes, the presence of multi-phases indicated the transition between two phases, such as between the FCC and the hexagonal or between the hexagonal and the lamellar phases. In addition, the phase transition of samples with lower molecular weight was more effectively controlled under the same conditions (temperature and additive concentration). The phase behavior trends of P65-5mS complex differed from those of P105- and P85-5mS complexes. On the other hand, a FCC phase was not observed for P65-5mS complex, but an isotropic phase existed between two different phases at varying temperatures. Although the formation of the isotropic phase after the appearance of the Bragg peak was unexpected, a different Bragg peak appeared once the temperature was raised. The ordered phase of P65-5mS complex continued with an isotropic phase, which highlights the different phase behavior of P65-5mS complex when compared to those of P105- and P85-5mS complexes. A schematic representation of the phase behaviors of P65-, P85-, and P105-5mS complexes is shown in Figure 6. The phase behaviors of the Pluronic block copolymers-5mS complexes can be understood by the coupled effect due to the simultaneous response of a geometrical conformation of the Pluronic block copolymer and the increase in the 5mS solubility depending on the temperature increases.

Although we expected that molecules with identical substances and mass fraction would undergo the same phase transitions, our results show that the phase behavior of P65 is an exception, as it showcased the unique CLL phase transition, where the solubility of 5mS increased with the temperature (5mS in the P65-5mS complex dissociates into water [17,18]), the increase of the 5mS solubility contributes to the decrease of the hydrophobicity of the P65-5mS complex. Considering the increase in the hydrophobicity of the PEO blocks of P65 by increasing the temperature, the hydrophobicity of the P65-5mS complex is determined by the coupled effect between the increasing hydrophobicity of PEO blocks and the decreasing hydrophobicity due to the 5mS dissociation in the complex. At a certain temperature region (where the decrease of the hydrophobicity due to the 5mS dissociation is more dominant), the P65-5mS complex has insufficient hydrophobicity to maintain an ordered phase, leading to an order to isotropic phase transition. On the other hand, the phase behaviors of P105- and P85-5mS complexes featured multi-phase transition processes that of P65-5mS complex showed an isotropic phase instead. The difference in the phase behaviors of P105-, P85-, and P65-5mS complexes is due to the momentums of the conformational entropy, which changed according to the geometrical conformation of the molecule. Therefore, even if the amphiphilic molecules have identical molecular chain and mass fraction, differences in the molecular weight (length of the chain) lead to changes in the geometrical conformation, which ultimately affect the phase behaviors. The study revealed that the P65-5mS complex preferably adopts a specific geometrical conformation with a relatively single phase despite its high concentration of 40 wt % in the sample. Furthermore, the phase behavior of P65-5mS shows that the phase transition of the block copolymer does not always depend on its molecular weight.

## 4. Conclusions

We report the investigation of the phase behaviors of complexes of Pluronic P105, P85, and P65 block copolymers with 5mS additive by visual inspection, POM measurements, and SAXS experiments. The three investigations successfully defined the phase behaviors of P105-, P85-, and P65-5mS complexes. The selection of the Pluronic block copolymers was based on their hydrophilic mass fraction, as the phase transitions of amphiphilic block copolymers with 0.5 mass fraction of the hydrophilic part can be sensitively controlled. It was expected that P105-, P85-, and P65-5mS complexes would have similar phase behaviors, however, our results showed that the phase behaviors were different, and were critically affected by the molecular weight. In the case of the P105- and P85-5mS complexes, ordered phases were observed over the entire experimental temperature range, whereas P65-5mS complex alternated between disordered and ordered phases. The phase behaviors of P105- and P85-5mS complexes consisted of the FCC, hexagonal, and lamellar phases, whereas those of P65 consisted of the isotropic, hexagonal, isotropic, and lamellar phases. The phase behaviors were strongly reflected by the visual inspections and POM measurements of the samples. For the P105- and P85-5mS complexes, the ordered phases correlated with the observed gel type; however, for the P65-5mS complex, which exhibits isotropic, hexagonal, isotropic, and lamellar phases, the visual inspection revealed gel- and fluidic phases. Therefore, we conclude that the energy of the molecule with the low molecular weight is insufficient to overcome the energy barrier for transition into an ordered phase, resulting in a reversal to the isotropic phase [35]. Molecules with low molecular weights are less prone to adopt a specific geometry than those with high molecular weights. The phase behavior phenomena are expected to find application in optical nanodevices such as optical nanosensors or nanoelectronics and nanotemplates by taking advantage of the sensitivity of the system to changes in the temperature.

## Data Availability

The data presented in this study are available on request from the corresponding author.
